# Early Onset Intrauterine Growth Restriction in a Mouse Model of Gestational Hypercholesterolemia and Atherosclerosis

**DOI:** 10.1155/2014/280497

**Published:** 2014-09-10

**Authors:** Dolores Busso, Lilian Mascareño, Francisca Salas, Loni Berkowitz, Nicolás Santander, Alonso Quiroz, Ludwig Amigo, Gloria Valdés, Attilio Rigotti

**Affiliations:** ^1^Departamento de Nutrición, Diabetes y Metabolismo, Escuela de Medicina, Facultad de Medicina, Pontificia Universidad Católica de Chile, Marcoleta 367/Interior, 4° Piso, 8330024 Santiago, Chile; ^2^Departamento de Gastroenterología, Escuela de Medicina, Facultad de Medicina, Pontificia Universidad Católica de Chile, Marcoleta 367/Interior, 4° Piso, 8330024 Santiago, Chile; ^3^Departamento de Nefrología y Centro de Investigaciones Médicas, Escuela de Medicina, Facultad de Medicina, Pontificia Universidad Católica de Chile, Lira 44, 2° Piso, 8330024 Santiago, Chile; ^4^Centro de Nutrición Molecular y Enfermedades Crónicas, Escuela de Medicina, Facultad de Medicina, Pontificia Universidad Católica de Chile, Avenida Bernardo O'Higgins 340, 8331150 Santiago, Chile

## Abstract

The susceptibility to develop atherosclerosis is increased by intrauterine growth restriction and prenatal exposure to maternal hypercholesterolemia. Here, we studied whether mouse gestational hypercholesterolemia and atherosclerosis affected fetal development and growth at different stages of gestation. Female LDLR KO mice fed a proatherogenic, high cholesterol (HC) diet for 3 weeks before conception and during pregnancy exhibited a significant increase in non-HDL cholesterol and developed atherosclerosis. At embryonic days 12.5 (E12.5), E15.5, and E18.5, maternal gestational hypercholesterolemia and atherosclerosis were associated to a 22–24% reduction in male and female fetal weight without alterations in fetal number/litter or morphology nor placental weight or structure. Feeding the HC diet exclusively at the periconceptional period did not alter fetal growth, suggesting that maternal hypercholesterolemia affected fetal weight only after implantation. Vitamin E supplementation (1,000 UI of *α*-tocopherol/kg) of HC-fed females did not change the mean weight of E18.5 fetuses but reduced the percentage of fetuses exhibiting body weights below the 10th percentile of weight (HC: 90% vs. HC/VitE: 68%). In conclusion, our results showed that maternal gestational hypercholesterolemia and atherosclerosis in mice were associated to early onset fetal growth restriction and that dietary vitamin E supplementation had a beneficial impact on this condition.

## 1. Introduction

In the last decades, the influence of the intrauterine development on the susceptibility to cardiovascular disease has been demonstrated both in humans and in experimental animal models. David Barker, pioneer in describing fetal early environmental programming, described how undernutrition* in utero* can alter human fetal growth and induce permanent changes in the body's structure, function, and metabolism that can lead to coronary heart disease later in life [1]. Additional studies showed that, beyond a caloric restriction, other intrauterine suboptimal conditions, such as excessive nutrient availability or exposure to pollutants, alcohol, or nicotine, can also increase the susceptibility of the adult offspring to cardiovascular disease (reviewed in [2]).

Maternal adverse conditions can affect embryos at different stages of gestation, even before implantation. In fact, mammalian preimplantation embryos from different animal models, ranging from mice to sheep, are particularly sensitive to environmental factors during the periconceptional period. Exposure of blastocysts to suboptimal conditions both* in vivo* (i.e., maternal undernutrition) and* in vitro* (i.e., inefficient culture conditions) is associated with the onset of cardiovascular dysfunction in adult life [3].

The most common underlying cause of ischemic cardiovascular disease is atherosclerosis, a silent and progressive pathologic condition that originates very early in life, even during development in the womb, characterized by the accumulation of lipids, lipoperoxidation products, and macrophages in arterial vessels [4]. During pregnancy, maternal high plasma cholesterol levels may promote fetal atherosclerosis. In humans, fatty streaks and intimal thickening are higher in fetuses from hypercholesterolemic mothers [4, 5]. Although the human term placenta is impermeable to the atherogenic low density lipoproteins (LDL), the existing correlation between maternal and fetal plasma cholesterol levels before the sixth month of gestation suggests that maternal high levels of plasma cholesterol at early to midpregnancy may directly promote lesion formation in the fetus [4]. Hypercholesterolemia during pregnancy increases maternal lipid peroxidation, which is transmitted to the embryo via the placenta, suggesting that abnormal cholesterol metabolism can also have indirect effects on the placenta and fetus, that is, oxidative stress [6]. Indeed, maternal hypercholesterolemia associates with umbilical vein endothelial dysfunction due to arginase and eNOS signaling imbalance [7]. In humans, suboptimal fetal growth is one of the risk factors for atherosclerosis: weighing less than the 10th percentile of the population or being born preterm increases the development of endothelial dysfunction and preclinical atherosclerosis in young adults [8]. Also, fetal growth restriction in rats leads to the early appearance of atherosclerosis in adult offspring [9].

Progress in understanding fetal programming of human atherosclerosis has been slowed by the high genetic and environmental heterogeneity among individuals and by the difficulty of accessing noninvasively images from aortas of developing fetuses. In addition, the requirement of cholesterol to sustain appropriate fetal development [10] has hindered the use of pharmacological treatments to reduce cholesterol in hypercholesterolemic pregnant women due to their possible teratogenic effects. As in many other areas of biomedical research, animals have nevertheless proven valuable experimental models of fetal programming due to their genetic homogeneity, more controlled environment, accessibility to obtain fetuses at different developmental stages, and feasibility for interventions during gestation. A rabbit model established by the group of Napoli and Palinski provided the first straightforward evidence on the pivotal role of maternal hypercholesterolemia in atherosclerosis in the offspring [11]. This study also reported the involvement of oxidative stress in fetal atherosclerosis, as lesions were significantly reduced when pregnant females were fed with vitamin E-supplemented hypercholesterolemic diets. Other studies using rabbits showed that near term fetuses from high-cholesterol-fed dams exhibited 15–25% lower body weight than fetuses from chow-fed controls [12, 13].

LDL receptor deficient (LDLR KO) [14] and apolipoprotein E deficient (ApoE KO) [15] mice have been used to study fetal programming of atherosclerosis. LDLR KO mice fed with regular chow have a twofold increase in LDL cholesterol levels compared with wild-type mice, yet these mice only develop atherosclerosis when fed with a high-cholesterol diet that increases their plasma cholesterol above 300 mg/dl [16]. Recent studies using these mice have contributed to the understanding of some of the mechanisms implicated in the high susceptibility to atherosclerosis observed in the offspring of hypercholesterolemic pregnant females. Fetal exposure to maternal hypercholesterolemia induces changes in gene expression in different tissues (i.e., aorta and liver) that persist into adulthood [14, 17]. Interestingly, LDLR KO females fed with a western-type high fat/carbohydrate/cholesterol diet for 6 weeks before conception and during pregnancy give birth to smaller pups [18]. However, in both studies, using hyperlipidemic rabbits or mice, the onset of fetal growth abnormalities during intrauterine development has not been studied.

This work was aimed at analyzing the impact of periconceptional (until E5.5) versus gestational maternal hypercholesterolemia and atherosclerosis on fetal development at different stages of intrauterine development. This maternal condition was not associated with miscarriages or severe fetoplacental abnormalities. Fetal growth restriction was detected in mice from both sexes as a consequence of gestational but not periconceptional maternal hypercholesterolemia. Taking into account the low levels of vitamin E detected in mothers and newborns from human growth restricted pregnancies [19, 20] and the reduction of oxidative stress by vitamin E in placenta of hypercholesterolemic rabbits [21], we also analyzed whether dietary supplementation with vitamin E could prevent growth restriction in fetuses from HC-fed females.

## 2. Results

### 2.1. LDLR KO Fed with a Proatherogenic Diet Develop Hypercholesterolemia and Atherosclerosis in the Absence of Overweight or Infertility

**Figure 1:**
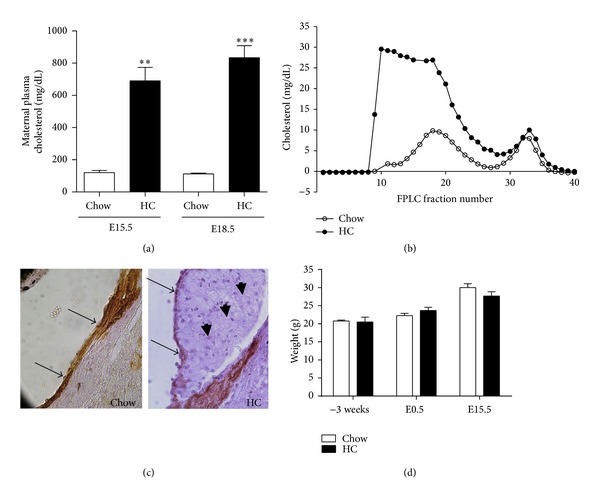
Gestational hypercholesterolemia and atherosclerosis in LDLR KO females fed with a HC diet. (a) Plasma cholesterol concentration was significantly higher in HC-fed females compared to chow-fed females at both E15.5 (**P < 0.05, *n* = 2 and *n* = 4 females, resp., Student's *t*-test) and E18.5 (***P < 0.001, *n* = 5 females in each group, Student's *t*-test). (b) Representative FPLC profiles of cholesterol content in lipoproteins in one chow- and one HC-fed pregnant female in E15.5. (c) Fatty streak in the aortic root of a HC-fed pregnant female in E15.5. Note the actin positive smooth cells (brown), the endothelial layer (arrows), and the foam cells in the subendothelial region (arrowheads) (magnification: 40x). (d) Feeding the HC diet did not induce overweight either before pregnancy or during pregnancy (*n* = 3 females in each group). Mean ± SEM.

LDLR female mice fed with a proatherogenic, high-cholesterol (HC) diet containing 0.625% cholesterol, fat, and cholic acid diet for 3 weeks were as fertile as females fed with a chow diet containing 0.02% cholesterol, as 4/5 (80%) of these hypercholesterolemic females with a vaginal plug became pregnant versus 5/6 (83%) in the control group. Feeding the HC diet before and during pregnancy induced severe hypercholesterolemia, essentially due to accumulation of non-HDL lipoproteins, in the endogenously hypercholesterolemic LDLR KO females [22] (7- and 8-fold at E15.5 and E18.5, resp.) (Figures 1(a) and 1(b)). This dyslipidemia led to the development of atherosclerotic lesions in the maternal aortic root as shown by immunohistochemical analyses (Figure 1(c)). No additional signs of adverse health conditions or body weight differences were detected in the diet-manipulated compared to the chow-fed LDLR KO group (Figure 1(d)).

### 2.2. LDLR KO Mice with Gestational Hypercholesterolemia and Atherosclerosis Exhibit Fetal Growth Restriction

**Figure 2:**
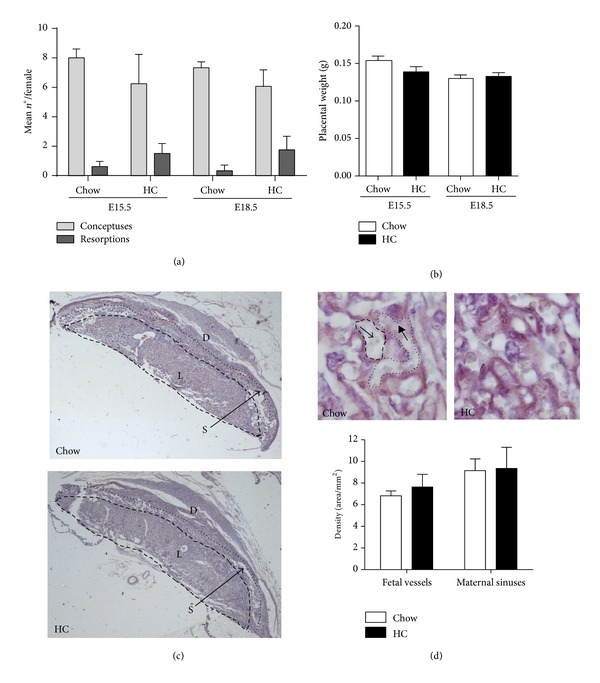
LDLR KO females fed with a HC diet have normal placental development. (a) The mean numbers of conceptuses and resorptions per female retrieved at E15.5 and E18.5 were similar in LDLR KO chow- and HC-fed pregnant females (*n* = 3 litters from each group). (b) Placenta from hypercholesterolemic pregnancies had similar weights compared to those from control pregnancies at E15.5 and E18.5. (c) Histological analyses of E15.5 placenta showed similar tissue organization and distribution of the different placental structures: labyrinth (L), spongiotrophoblast (S), and decidua (D) in control and hypercholesterolemic placenta (*n* = 3 per group) (magnification: 8x). (d) Analysis of the vascular organization in the labyrinth in control and HC pregnancies (*n* = 3 placentas from each group). Upper panel: representative immunohistochemistry showing anticytokeratin positive syncytiotrophoblasts marking the interface between maternal and fetal circulations (magnification: 1,000x). Lower panel: quantification of the mean areas per mm^2^ labyrinth occupied by fetal vessels (surrounded by dashed line) containing large, immature red blood cells (arrow) and maternal sinuses (surrounded by dotted line) containing mature, enucleated red blood cells (arrowhead). Mean ± SEM.

Neither the litter size nor the mean number of resorptions differed between chow-fed and HC-fed pregnant LDLR KO females (Figure 2(a)). Placental gross morphology and size (not shown) and placental weight (Figure 2(b)) were also similar in both groups at E15.5 and E18.5. The distribution of tissues conforming the three different layers of the mouse placenta, decidua, spongiotrophoblast, and labyrinth was normal in females from both groups at E15.5 (Figure 2(c)) and E18.5 (not shown). No defects in the labyrinth organization and the distribution of vascular exchange areas within placenta from hypercholesterolemic and atherosclerotic females were detected in histological sections from E15.5 placenta, as the mean area occupied by maternal sinuses and fetal capillaries was similar in placenta from chow- and HC-fed groups (Figure 2(d)).

**Figure 3:**
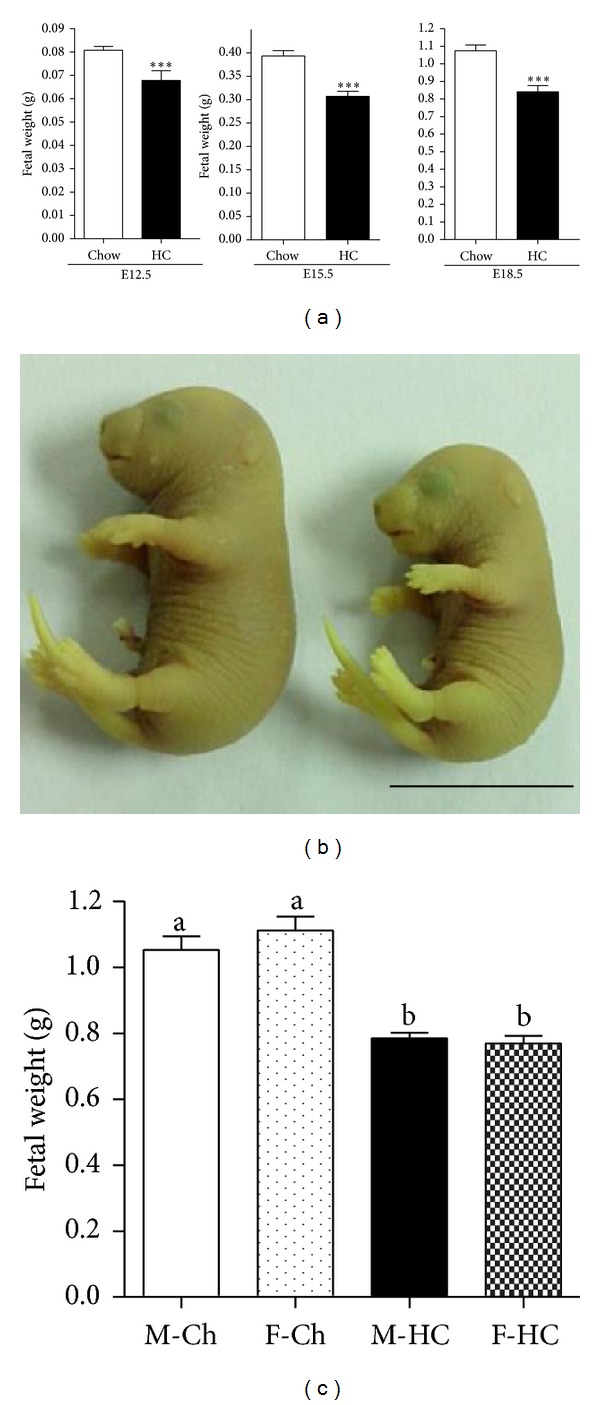
Fetal intrauterine growth restriction is observed in LDLR KO mice fed with a HC diet. (a) Fetuses retrieved from hypercholesterolemic pregnant females weighed significantly less than fetuses from control mice at the three stages analysed (***P < 0.001) (E12.5, E15.5, and E18.5, *n* = 43, 24, and 10 fetuses in control females, and *n* = 17, 19, and 31 fetuses in HC-fed females, resp.). (b) Representative picture of a normal fetus obtained from a chow-fed female and one growth restricted fetus obtained from a HC-fed female fixed in Bouin. (c) Fetal weight in E18.5 male and female fetuses from control and HC groups (*n* = 7 to 10 fetuses in each group). Mean ± SEM.

Despite the normal placental growth and morphological parameters observed in pregnancies occurring in LDLR KO mice with hypercholesterolemia and atherosclerosis, the mean body weight of fetuses harvested from these females was lower than that of fetuses from chow-fed mice at the three gestational ages analyzed (Figures 3(a) and 3(b)). Fetal sex discrimination by PCR amplification of* smcx* and* smcy* alleles showed that fetal growth restriction was similar in both sexes in the HC-fed group (Figure 3(c)).

### 2.3. Periconceptional Exposure to Hypercholesterolemia and Atherosclerosis Does Not Affect Fetal Growth

To determine whether exposure to maternal hypercholesterolemia exclusively during the periconceptional period had an effect on fetal growth at later stages of intrauterine development, LDLR KO females were fed with the proatherogenic HC diet from 3 weeks previous to conception until implantation at E5.5 and then fed with regular chow diet. Plasma cholesterol levels from females exposed to the HC diet periconceptionally were already normalized at E15.5 [115 ± 25 mg/dl in periconceptional HC-fed LDLR KO females (*n* = 2 females) versus 108 ± 30 mg/dl in control chow-fed LDLR KO females (*n* = 2 females), *P* = 0.88 Student's *t*-test]. E15.5 fetuses retrieved from dams periconceptionally fed with the HC diet weighed similar to fetuses from chow-fed mice [382 ± 22 mg (*n* = 11 fetuses) versus 393 ± 22 mg (*n* = 24 fetuses), resp., *P* = 0.58 Student's *t*-test]. These results indicated that periconceptional maternal hypercholesterolemia in mice does not impact fetal growth.

### 2.4. Dietary Vitamin E Supplementation Reduces the Proportion of Growth Restricted Fetuses in Pregnancies with Gestational Hypercholesterolemia and Atherosclerosis

**Figure 4:**
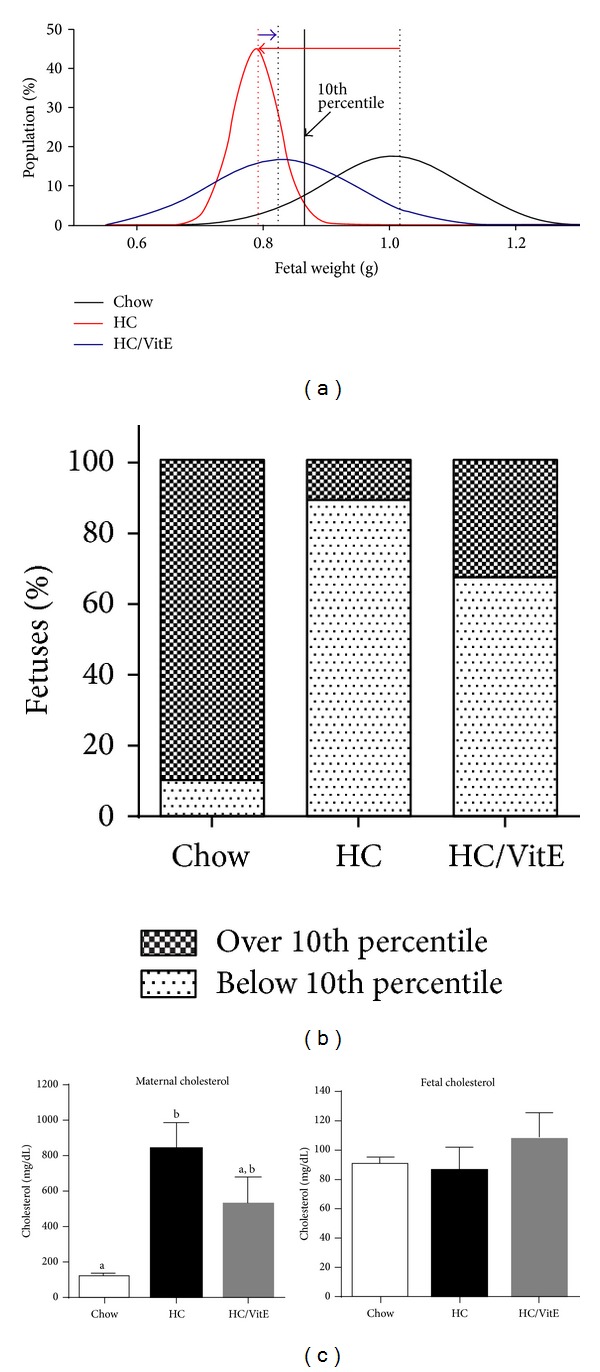
Effect of vitamin E supplementation on fetal growth restriction induced by maternal hypercholesterolemia in HC-fed LDLR KO mice. (a) Gauss normal curves for E18.5 fetal weights in females fed with chow (black, *n* = 58 fetuses, *r* ^2^ = 0.86), HC (red, *n* = 47 fetuses, *r* ^2^ = 0.91), or HC/VitE (blue, *n* = 38 fetuses, *r* ^2^ = 0.89) diets. The black continuous line represents the 10th percentile of WT fetal weight (865 mg); dashed lines indicate the mean weight for each group. The difference between weights from the HC- and chow-fed groups and HC- and HC/VitE-fed groups is represented by red and blue arrows, respectively. (b) Percentage of growth restricted fetuses showing an increase in fetuses over the 10th precentile in the HC/VitE group (32%) compared to the HC group (10%). (c) Left panel: high plasma cholesterol in HC-fed females was partially reduced by vitamin E supplementation (different lettering indicates significance at *P* < 0.05, one-way ANOVA, *n* ≥ 3 females). Right panel: fetal plasma cholesterol was similar in females receiving chow, HC, or HC/VitE diets (*n* ≥ 3 pools from 2 fetuses).

LDLR KO females were fed with chow, HC, or vitamin E-supplemented HC (HC/VitE) diets from 3 weeks before conception until E18.5, when fetuses were retrieved. Fetal weights displayed frequency distribution curves with the expected Gaussian distribution (Figure 4(a)) [23]. As observed previously (in Figure 3), maternal HC-feeding of LDLR KO females resulted in a significant reduction in the mean fetal weight (1.01 ± 0.02 g control (*n* = 58 fetuses) versus 0.79 ± 0.01 g HC (*n* = 38 fetuses), *P* < 0.001 Student's *t*-test). This defect can be appreciated in Figure 4(a) by the displacement of the frequency distribution curve to the left (see red arrow). In the HC-fed group, 90% of the fetuses exhibited weights that were under the 10th percentile of the control chow-fed population (Figure 4(b)). Vitamin E supplementation of HC-fed dams did not affect the mean weight of fetuses compared to the HC untreated group [0.82 ± 0.02 g HC/VitE (*n* = 47 fetuses), *P* = 0.147 Student's *t*-test] (small displacement of curves indicated by blue arrow) (Figure 4(a)). However, the percentage of fetuses weighing less than the 10th percentile of the control group was reduced from 90% in the untreated HC-fed group to 68% in the HC/VitE group (Figure 4(b)). The difference in the proportions of normal versus growth restricted fetuses in the HC-fed untreated (4 versus 34) and vitamin E- supplemented (15 versus 32) groups reached statistical significance (*P* = 0.0207, Fisher's exact test).

Vitamin E supplementation had an impact on plasma cholesterol levels in HC-fed dams, although this reduction did not reach statistical significance compared to HC-fed females [118 ± 6 mg/dl chow (*n* = 3 females), 844 ± 139 mg/dl HC (*n* = 4 females), and 527 ± 147 mg/dl HC/VitE (*n* = 3 females)] (Figure 4(c), left panel). Neither HC nor HC/VitE diets affected the levels of fetal plasma cholesterol at E18.5 [90 ± 5 mg/dl control (*n* = 3), 86 ± 16 mg/dl HC (*n* = 4), and 107 ± 18 mg/dl HC/VitE (*n* = 3); in this case each *n* corresponds to a pool of 2 fetuses] (Figure 4(c), right panel), which is consistent with the lack of association between maternal and fetal cholesterol levels in late pregnancy observed in humans [4].

## 3. Materials and Methods

### 3.1. Animals

LDLR KO mice (B6.129S7-Ldlrtm1Her/J) were originally purchased from The Jackson Laboratory. Animals were housed in a temperature- and light-controlled room. Protocols were conducted in agreement with the National Research Council (NRC) Publication Guide for the Care and Use of Laboratory Animals (8th edition, 2011, National Academy of Sciences, USA). These studies were approved by the Ethics Committee for Animal Welfare from the School of Medicine of the Pontifical Catholic University of Chile. Six-week-old LDLR KO females were fed with a standard* chow diet* [0.02% cholesterol (Prolab RMH3000, Labdiet)],* high-cholesterol diet* [0.625% cholesterol 1 : 1 mix of chow diet and 57BB diet atherogenic diet], or a* high-cholesterol and vitamin E-supplemented diet* [containing 0.625% cholesterol and 1,000 UI of vitamin E/kg (1 : 1 mix of the 57BB atherogenic diet and Prolab 5P00 diet containing 2,000 IU vitamin E/kg, Labdiet)]. After 3 weeks receiving each diet, two females were housed with one male and the presence of vaginal plug was checked daily within the first hour of the light cycle. When the vaginal plug was detected, embryonic day 0.5 (E0.5) was recorded. All diets used before conception were continued during pregnancy until the collection of the embryos, except in the protocol where the impact of periconceptional maternal hypercholesterolemia was analyzed, in which females were fed with the HC diet until 5.5 and then received standard chow until embryo collection.

At E12.5, E15.5, or E18.5 pregnant dams were deeply anesthetized with a mix of ketamine: xylazine (100 : 10 mg per kg body weight) and blood was collected from the vena cava with a heparinized syringe. The dams were euthanized and the embryos and extraembryonic tissues were retrieved and kept on ice. Each embryo and placenta were weighed before fixation in Bouin's fixative. Yolk sacs were used for sexing by PCR. At E18.5, fetal blood was collected after decapitation and blood pooled from 3 fetuses was used for cholesterol determinations.

### 3.2. Fetal Sex Determination by PCR

Individual sexing of embryos was performed by PCR as described elsewhere [24]. The following primers, F: 5′-CCGCTGCCAAATTCTTTGG-3′ and R: 5′-TGAAGCTTTTGGCTTTGAG-3′, were used to amplify bands of different sizes corresponding to the* smcx/y* gene in X and Y chromosomes. Samples were amplified using the GoTaq (Promega) PCR kit, following manufacturer instructions with the following PCR program: 2 minutes at 95°C, 35 cycles of 30 s at 95°C, 30 s at 58°C, 30 s at 72°C, and a final extension of 10 minutes at 72°C. Amplicons were resolved in a 2% agarose gel and the presence of one or two bands indicated female or male, respectively.

### 3.3. Cholesterol Determination

Plasma cholesterol concentration was determined using a standard enzymatic method reported previously [25]. Samples were incubated for 30 minutes at 37°C with 0.5 M Tris, pH 7.6, 50 mM phenol, 50 mM 4-chlorophenol, 1% Triton X-100, 0.37% sodium cholate, 0.04% 4-aminoantipyrine, 0.35 U/mL cholesterol esterase, 0.1 U/mL cholesterol oxidase, 1 U/mL peroxidase, and 490 nm absorbance.

### 3.4. Lipoprotein Chromatographic Separation

Plasma samples were subjected to chromatographic separation using a Superose-6 molecular exclusion column (GE Healthcare Life Sciences) and elution buffer (150 mM NaCl, 1 mM EDTA, pH 7.8) at a constant flux of 9 ml/h. Cholesterol content in each fraction was determined as described above.

### 3.5. Histological and Immunohistochemical Analyses

Bouin-fixed tissues were dehydrated and embedded in paraffin. Tissue sections (8 *µ*m) were stained with hematoxylin and eosin or used for immunohistochemical procedures. For immunolocalization, sections were subjected to antigen retrieval using hot citrate and then incubated with commercial rabbit antibodies against smooth muscle actin (1 : 200, Sigma) or cytokeratin (1 : 200, Dako). After extensive washing, sections were incubated with appropriate secondary antibodies coupled to horseradish peroxidase (1 : 800, Sigma) and revealed with diaminobenzidine (Sigma). Negative controls included slides where the primary antibody was replaced by nonimmune rabbit serum.

### 3.6. Identification of Atherosclerotic Lesions

Whole hearts from pregnant dams were fixed and processed for immunohistochemical detection of smooth muscle actin as described above. Only sections that passed through the aortic root were considered, as turbulent flow promotes plaque formation in this part of the aorta. Foam cell infiltrates between the smooth muscle layer and the endothelium and positive staining of the endothelial cells indicated the presence of early atherosclerotic lesions.

### 3.7. Morphometric Analyses

Transversal sections of whole placenta containing the central arterial canal were immunostained with the anti-cytokeratin antibody to localize syncytiotrophoblast layers and identify maternal sinuses and fetal vessels. Fetal vessels were identified by the presence of an endothelium and enucleated red blood cells, whereas maternal sinuses were surrounded by syncytiotrophoblasts and contained mature, enucleated red blood cells. The area of all the fetal vessels or maternal sinuses in at least 3 different slides was quantified using ImageJ software (NIH, Bethesda). The occupancy of fetal and maternal areas in placenta was expressed as the mean of area of capillaries or sinuses per mm^2^ in the slide.

### 3.8. Statistical Analyses

Analyses were performed using GraphPad Prism 6 software. Data are presented as mean ± SEM. The significance of the differences between means was evaluated using Student's *t*-test or ANOVA and Tukey post hoc test. The 10th percentile was calculated as *R* = *P*∗(*n* + 1)/100, where *P* is 10 and *n* is the number of values in the chow group. The significance of the differences between proportions was analyzed using Fisher's exact test. Differences were considered significant at *P* < 0.05.

## 4. Discussion

In the present study we used a mouse model of pregnancy that exhibited high levels of plasma cholesterol and atherosclerosis, independent of overweight. Pregnancies in hypercholesterolemic and atherosclerotic females were characterized by normal numbers of fetuses and occasional resorptions, discarding a gross effect of this pathological metabolic condition on inhibition of implantation or abortion. The analysis of placental weight and morphology at two stages of late pregnancy, E15.5 and E18.5, indicated that the placenta grew and differentiated normally in HC-fed females. The labyrinth developed normally as well, as maternal and fetal vascular areas in this region were unaffected by maternal HC-feeding. Future studies will allow determining whether maternal hypercholesterolemia and atherosclerosis induce functional changes in placental nutrient and gas exchange.

A significant reduction in body weight was observed in fetuses from midpregnancy to preterm. This fetal growth restriction was already present as early as E12.5, a developmental stage when placenta achieves maturation in mice and when fetuses start growing exponentially. Whereas newborns or term fetuses from hypercholesterolemic rabbits and mice were previously shown to exhibit lower weights than control animals [13, 24], to our knowledge this is the first evidence describing fetal growth restriction associated with high maternal cholesterol levels during early stages of gestation.

As mentioned previously, preimplantation embryos can be susceptible to environmental stressors. Evidence from different experimental animal models showed that the exposure of embryos before implantation to adverse developmental conditions (i.e., undernutrition, overnutrition, and inflammation), both* in vitro* and* in vivo*, can not only hinder the embryonic quality and implantation success but also exert more subtle effects that are expressed later during intrauterine development or even during adulthood [26–28]. Programming of disease can occur as early as in oocytes and zygotes, by mechanisms including the aberrant methylation of genes involved in body homeostasis [29, 30], defective mitochondrial function [28, 31], and the inefficient generation of appropriately sized stem-cell lineages due to abnormal proliferation [32]. Our studies showed that exposure to maternal hypercholesterolemia exclusively before implantation was harmless to embryo growth, as LDLR KO females fed with the proatherogenic HC diet periconceptionally did not exhibit fetal growth restriction. However, the existence of possible effects of periconceptional HC-feeding on other embryonic parameters besides embryonic growth cannot be ruled out from our analyses.

Epidemiological studies first described in the late 1990s showed that intrauterine growth is more affected by maternal undernutrition in boys than in girls [33]. It has been hypothesized that male and female human fetuses adopt different strategies to overcome low nutrient availability; for example, male fetuses trade off visceral development* in utero* to protect somatic and brain growth, an adaptive beneficial response which can promote chronic metabolic diseases during adulthood [34]. Several gender-specific differences in fetal responses to other adverse maternal conditions have been reported in animal models [35–37]. However, in our studies fetal growth restriction induced by HC-diet maternal hypercholesterolemia and atherosclerosis was not gender-specific, as both male and female fetuses were lighter than fetuses from control chow-fed females.

Both maternal hypercholesterolemia and intrauterine growth restriction in humans have been independently linked to the early onset of subclinical atherosclerosis and a higher predisposition to cardiovascular disease in their offspring [21, 38]. However, a direct link between these two conditions is not clear because, on the one hand, women carrying fetuses with intrauterine growth restriction have lower, and not higher, total cholesterol and HDL-cholesterol concentrations [39] and, on the other hand, no significant differences have been found in newborn weights from normal and hypercholesterolemic pregnancies [6, 40].

Oxidative stress is considered one of the potential pathological insults given by maternal hypercholesterolemia on placenta and fetuses. Indeed, plasma from hypercholesterolemic women shows an increase in markers in both placenta and fetus [6]. Interestingly, oxidized LDL particles accumulate within the human term placenta in pregnancies undergoing early onset intrauterine growth restriction [41]. In animal experimental models, maternal antioxidant treatment has shown to enhance fetal growth and/or prevent growth restriction [21, 42]. In our studies, vitamin E antioxidant supplementation was not sufficient to increase the mean fetal weight in HC-fed females. However, the fact that this vitamin significantly reduced the proportion of growth restricted fetuses in HC-fed dams suggested that oxidative stress could be one of the mechanisms implicated in the pathogeny of fetal growth restriction in this model. Nonantioxidant vitamin E activities may also be implicated in the beneficial effect of this vitamin on fetal growth in HC-fed females. The beneficial effect of vitamin E on fetal growth could be mediated by the lowering of cholesterol. It has been shown recently that vitamin E can reduce cholesterol biosynthesis* in vitro* [43]. As well, vitamin E supplementation of rats and hamsters fed with atherogenic diets can reduce their plasma cholesterol levels [44, 45]. Given the trend in lowering plasma cholesterol observed in vitamin E-supplemented HC-fed LDLR KO females, further studies will be pursued to determine the possible beneficial effects of this vitamin on the maternal cholesterol metabolism in hypercholesterolemic mouse pregnancies. On the other hand, vitamin E has been shown to regulate molecular pathways controlling cell proliferation and viability [46] and could promote fetal growth in pregnant mice with hypercholesterolemia and atherosclerosis. Vitamin E can also enhance the release of vasodilator prostanoids from human endothelial cells from the aorta [47] and umbilical cord [48] and could favor placenta-fetal blood flow, increasing nutrient absorption. Although vitamin E has not been successful in reducing the risk of major cardiovascular events in humans [49], its supplementation could still be useful to prevent the fetal programming of atherosclerosis, as observed in the offspring of HC-fed rabbits [21]. Our future studies will determine whether vitamin E maternal supplementation has an impact on fetal programming of atherosclerosis in mouse offspring from HC-fed LDLR KO.

The detection of low birth weight in rabbit and mouse newborns from hypercholesterolemic pregnancies [12, 24], together with the results of this work where early fetal growth was observed in HC-fed LDLR KO dams, is in contrast to studies showing that fetuses from human mothers with high-cholesterol levels exhibit normal birth weights [6, 40]. Species-specific differences in the response of fetal growth to maternal hypercholesterolemia could explain the divergent results in animals and humans. In this regard, although the mice have been a useful experimental model of fetal programming due to the high genetic homogeneity and the possibility to test dietary and pharmacological interventions during pregnancy, their lipoprotein metabolism has fundamental differences with humans [50]. In humans, cholesterol is mainly transported in LDLs whereas in mice cholesterol is carried almost exclusively in HDLs, mainly due to the fact that mice lack cholesterol ester transfer protein (CETP), which transfers cholesterol from HDLs to LDLs. Among the various genetically modified mice that have contributed greatly to progress in the field of dyslipidemia [51], the LDLR KO mice exhibit a lipoprotein profile that is very similar to the human normal profile [52]. However, due to the rapid clearance of LDL cholesterol through the apoE receptor, which is present in mouse and not in human livers, the accumulation of LDL in plasma in LDLR KO mice is mild, and high-cholesterol diets need to be used to induce significant hypercholesterolemia [53]. These differences need to be considered when trying to interpret the results obtained using mice as models of human dyslipidemia.

One limitation of this study is that the atherogenic diet used in the study contains cholic acid, a component commonly added to cholesterol-enriched mouse diets in order to increase cholesterol absorption and induce atherosclerosis. In a recent publication, mice fed with a chow diet supplemented with cholic acid were used to establish a model of intrahepatic cholestasis of pregnancy [54]. Strikingly, in that study cholic acid supplementation was shown to affect lipid biosynthesis and transport in the fetoplacental unit and increase the susceptibility of the offspring to metabolic disease. Thus, the fact that high-cholesterol diets containing cholic acid can induce more than one pathogenic condition in pregnant female mice (e.g., hypercholesterolemia and intrahepatic cholestasis of pregnancy) needs to be kept in mind when interpreting the results in this model, particularly when analyzing fetal programming. Regarding fetal weight, this parameter was not affected by maternal cholic acid supplementation [54], suggesting that the main effect on fetal growth in our study is not due to cholic acid itself.

In summary, this study shows that, in LDL KO mice, diet-induced maternal hypercholesterolemia and atherosclerosis during pregnancy can negatively impact fetal growth. Fetal growth restriction associated with this maternal condition is characterized by an early onset and similar prevalence in both sexes. Vitamin E dietary supplementation has a beneficial effect, preventing growth restriction in a significant proportion of fetuses from HC-fed mice. Further studies in this mouse model will allow understanding the mechanisms explaining fetal growth restriction and the possible prevention of atherosclerosis in the offspring by maternal vitamin E supplementation. This knowledge could be useful in designing strategies aimed at preventing or reducing cardiovascular disease prenatally.
